# *Triatoma vitticeps* subcomplex (Hemiptera, Reduviidae, Triatominae): a new grouping of Chagas disease vectors from South America

**DOI:** 10.1186/s13071-017-2129-1

**Published:** 2017-04-13

**Authors:** Kaio Cesar Chaboli Alevi, Jader de Oliveira, Maria Tercília Vilela de Azeredo-Oliveira, João Aristeu da Rosa

**Affiliations:** 1 Departamento de Biologia, Instituto de Biociências, Letras e Ciências Exatas, Universidade Estadual Paulista “Júlio de Mesquita Filho”, Câmpus de São José do Rio Preto, Rua Cristovão Colombo 2265, 15054-000 São José do Rio Preto, SP Brazil; 2 Departamento de Ciências Biológicas, Faculdade de Ciências Farmacêuticas, Universidade Estadual Paulista “Júlio de Mesquita Filho”, Câmpus de Araraquara, Rod. Araraquara-Jaú km 1, 14801-902 Araraquara, SP Brazil

**Keywords:** *Triatoma vitticeps*, *Triatoma melanocephala*, Triatomini tribe

## Abstract

**Background:**

Triatomines have been grouped into complexes and subcomplexes based largely on morphological and geographical distribution. Although these groupings are not formally recognised as taxonomic ranks, they are likely monophyletic. However, recent studies have demonstrated that some subcomplexes from South America did not form monophyletic groups, and reorganisations have been suggested. One suggested reorganisation is to exclude *Triatoma vitticeps*, *T. melanocephala*, and *T. tibiamaculata* from the *T. brasiliensis* subcomplex. However, *T. vitticeps* and *T. melanocephala* exhibit several similar characteristics, including morphologic, cytogenetic, and phylogenetic features, a factor which supports the creation of a new subcomplex. Thus, this study aimed to describe the *T. vitticeps* subcomplex.

**Results:**

*T. vitticeps* and *T. melanocephala* are sister species and share a phylogenetic relationship, several similar morphological characteristics, the same composition of constitutive heterochromatin (Xs CG-rich and Y AT-rich), the same karyotype (2n = 20A + X_1_X_2_X_3_Y), and the same meiotic behaviour during spermatogenesis. Based on karyosystematics, for example, the *T. vitticeps* subcomplex may differ from all of the other subcomplexes from South America, as well as from the Rhodniini tribe and the genus *Panstrongylus*. We argue that the case of agmatoploidy involving the X chromosome was responsible for the karyotype divergence of this subcomplex in relation to the other South America subcomplexes.

**Conclusions:**

Based on the phenotypic characteristics (morphology) and genotypes (cytogenetics and molecular features), we propose the creation of the monophyletic *T. vitticeps* subcomplex, which we believe is distinct from all other subcomplexes from South America.

## Background

Chagas disease is a potentially life-threatening illness caused by the protozoan *Trypanosoma cruzi* (Chagas, 1909), which is most commonly distributed in endemic areas of 21 Latin American countries. The disease is most frequently transmitted to humans through contact with faeces of triatomines. It is estimated that about 6 million to 7 million people are infected worldwide, most of whom reside in Latin America [1].

Chagas disease vectors belong to the order Hemiptera, the suborder Heteroptera, the family Reduviidae and, the subfamily Triatominae [2]. This subfamily is composed of 151 species distributed across 18 genera and five tribes [2–5], and all species (nymphs and adults of both sexes) are considered to be potential vectors of *T. cruzi*.

Based mainly on morphological and geographical distribution, these vectors have been grouped into complexes and subcomplexes [6–11]. Although these groupings are not formally recognized as taxonomic ranks and, thus do not necessarily represent natural groups, Justi et al. [12] propose that they are likely to be monophyletic: once the relationships between vector species are known, information about a species may be reliably extrapolated to other closely related species [13].

The species of the Triatomini tribe have been grouped into three groups, eight complexes, and eight subcomplexes [11]; the main species groups are *Triatoma rubrofasciata* (present mainly in North America and the Old World) and *T. infestans* (present in South America). South American triatomines were initially grouped into the *T. infestans* complex and the *T. brasiliensis*, *T. infestans*, *T. matogrossensis*, *T. maculata*, *T. rubrovaria*, and *T. sordida* subcomplexes [11]. However, several studies have demonstrated that the *T. brasiliensis* [12, 14–16], *T. matogrossensis* [17–19], *T. rubrovaria* [16, 17], and *T. sordida* subcomplexes [16, 19] do not form monophyletic groups, and reorganizations of the subcomplexes have been suggested [14–16, 20].

As part of these suggested reorganisations, Alevi et al. [14] and Gardim et al. [16] suggest that *T. vitticeps* (Stal, 1859), *T. melanocephala* Neiva and Pinto, 1923, and *T. tibiamaculata* (Pinto, 1926) should be excluded from the *T. brasiliensis* subcomplex. In some studies, it has been argued that these species lack a subcomplex [21, 22]. However, phylogenetic analyses detected a relationship between *T. tibiamaculata* and *Panstrongyus megistus* (Burmeister, 1835), which have been found to be sister species [12, 16, 23]. Recently, Justi et al. [19] argued that *T. tibamaculata* is a member of the clade *megistus*, along with other species of *Panstrongylus* [+ *Nesotriatoma bruneri* (Usinger 1944)].

Meanwhile, *T. vitticeps* and *T. melanocephala* were not grouped into any new subcomplexes, since these species do not share phenotypic and genotypic characteristics with the triatomine subcomplexes from South America [14, 15, 24–26]. However, these species exhibit several similar characteristics, including morphological [27], cytogenetic [14], and phylogenetic [19] features, similarities which support the creation of a new subcomplex. Thus, this study aimed to describe, for the first time, the *T. vitticeps* subcomplex, highlighting the main phenotypic and genotypic characteristics that support the grouping of these species and how this subcomplex is distinct from the others present in South America.

## Methods

Ten adult males of each species of the new subcomplex were used for cytogenomic analysis. The species considered herein were *T. vitticeps* [geographic origin: Guarapari, Espírito Santo, Brazil (Coordinates: 20°39'01.41478"S, 40°30'25.29000"W)] and *T. melanocephala* [geographic origin: Bom Jesus da Serra, Bahia, Brazil (Coordinates: 14°22'04.46160"S, 40°30'52.55281"W), Jequié, Bahia, Brazil (Coordinates: 13°51'03.75834"S, 40°04'52.22281"W), and Poções, Bahia, Brazil (Coordinates: 14°31'01.94880"S, 40°22'43.37040"W)]. The specimens were provided by the Triatominae Insectarium within the Department of Biological Sciences in the College of Pharmaceutical Sciences at Sao Paulo State University’s Araraquara campus (FCFAR/UNESP), São Paulo, Brazil. The seminiferous tubules were torn apart, crushed, and fixed on slides in liquid nitrogen. The cytogenomic technique of CMA_3_/DAPI banding was then applied [28], with the modifications offered by Severi-Aguiar et al. [29] for differentiating the regions of heterochromatin rich in AT and CG. The biological material was analysed using an Olympus BX-FLA fluorescence microscope.

## Results and discussion

**Fig. 1:**
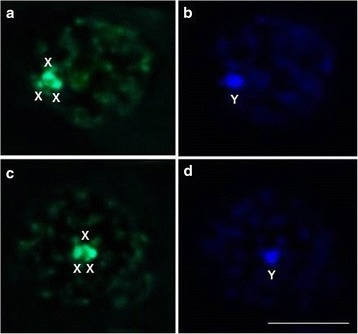
Composition of constitutive heterochromatin in *T. vitticeps* (**a**, **b**) and *T. melanocephala* (**c**, **d**). Note that the X chromosomes are rich in CG (**a**, **c**) and the Y chromosome is rich in AT (**b**, **d**). X: X chromosomes, Y: Y chromosome. *Scale-bar*: 10 μm

Both species presented the same composition of constitutive heterochromatin: X chromosomes rich in CG (Fig. 1a, c) and Y chromosome rich in AT (Fig. 1b, d), as initially observed by Severi-Aguiar et al. [29] in a study on *T. vitticeps* (initial prophases were used because the decondensed chromatin allows the labeling with fluorochromes to be more specific. Although Bardella et al. [22] have observed a small difference using CMA_3_/DAPI in *T. vitticeps*, we consider that size and compaction of the holocentric chromosomes in the metaphases may have made it difficult to interpret the results). In addition to this similarity, these species also share several morphological characteristics [27], and they exhibit the same 2n = 24 karyotype (20A + X_1_X_2_X_3_Y) [14], the same meiotic behavior during spermatogenesis [29, 30], and the possible ability to produce natural hybrids (personal communication), all of which supports the grouping of these species into a *T. vitticeps* subcomplex (the *T. vitticeps* name was chosen based on *T. vitticeps* being the first species of the subcomplex described in the literature).

Cytotaxonomy and karyosystematics are important tools for determining the taxonomy of triatomines [14, 15, 31, 32]. For example, the karyotype analysis of the species within this *T. vitticeps* subcomplex is what distinguishes these species from all of the other South American subcomplexes, which have 2n = 22 (20A + XY) chromosomes [33, 34], from all of the species of the Rhodniini tribe (2n = 22) [35], and from the species of the genus *Panstrongylus* (2n = 21 or 23) [33].

**Fig. 2:**
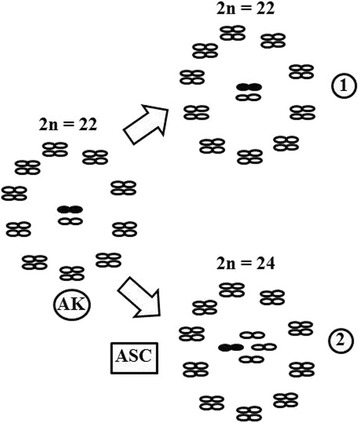
Karyotype evolution of the South American subcomplexes. (1) Subcomplexes that remained with the same number of chromosomes from the ancestral karyotype (*T. brasiliensis*, *T. infestans*, *T. matogrossensis*, *T. maculata*, *T. rubrovaria* and *T. sordida*). (2) *T. vitticeps* subcomplex. Note that the species in this subcomplex have undergone two agmatoploidy events in the X chromosome. *Abbreviations*: AK, ancestral karyotype; ASC, agmatoploidy in sex chromosome

In a recent phylogenetic study based on geological events, Justi et al. [19] suggested that *T. vitticeps* and *T. melanocephala* reached the Atlantic coast by dispersal and diversified prior to the Northern Andean uplift (23–10 Ma), an event which separated *T. maculata* from the other members of the *T. infestans* group. Based on this argument, and considering the fact that the ancestral karyotype of triatomines is 2n = 22 (20A + XY) [36, 37], we suggest that case of agmatoploidy involving the X chromosome was responsible for the karyotype divergence of this subcomplex in relation to the other South American subcomplexes (Fig. 2). Moreover, we argue that this was a unique event in the karyotype evolution of the *Triatoma* from South America, because, in addition to *T. melanocephala* and *T. vitticeps*, the only species that also presents fragmentation of the X chromosome is *T. tibiamaculata* 2n = 23 (20A + X_1_X_2_Y) [38]. However, the analysis provided by Justi et al. [19] allows us emphasise that this species inherited this number of chromosomes from the common ancestor shared with the *Panstrongylus* (a genus in which most of the species also have 23 chromosomes).

## Conclusion

Based on the phenotypic characteristics (morphology) and genotypes (cytogenetics and molecular features) that define these species, we propose the creation of the monophyletic *T. vitticeps* subcomplex, one which believe is distinct from all other subcomplexes from South America.
